# Exotic Viral Diseases: A Global Guide

**DOI:** 10.3201/eid0910.030490

**Published:** 2003-10

**Authors:** Michael Bell

**Affiliations:** * Centers for Disease Control and Prevention, Atlanta, Georgia, USA

Exotic Viral Diseases: A Global Guide by Berger et al. is a small, soft-cover, 252-page handbook organized by disease or disease group. It addresses 55 viral pathogens, including a few broad groupings, such as “New World hantaviruses.”

Because of increasing travel, which is eroding geographic barriers to disease transmission, and because of the emergence and reemergence of uncommon infectious diseases, frontline clinicians are increasingly more likely to encounter patients with exotic viral infections. The arrival of West Nile virus and monkeypox virus in North America demonstrates the potential for importation of unusual viral pathogens.

An 8-page overview of the assessment and evaluation of febrile viral syndromes precedes a series of four tables listing syndrome complexes, animal reservoirs, infectious vectors, and routes of infection. The tables are followed by sections describing each pathogen in alphabetical order. Each section includes common and generic designations for the virus, reservoirs, vectors, modes of transmission, incubation periods, “clinical hints,” “typical therapy,” and geographic distribution of the disease. Concise subsections provide general information, describe the clinical presentation and course of the illnesses, and recommend diagnostic procedures and laboratory biosafety levels. Sections end with a list of additional reading, ranging from 3 to 22 citations, including journal articles, book chapters, and electronic citations. In addition to the tables noted above, 10 charts are distributed throughout the sections, demonstrating disease incidence during recent decades. The book is indexed and has a 10-page appendix of drugs and vaccines and a 5-page appendix of diagnostic tests. It has no illustrations. An accompanying mini-CD ROM contains the same material as the printed edition.

Though the handbook’s coverage of topics is superficial, its format makes this a useful quick reference, for example, as a reminder for clinicians assembling differential diagnosis lists for a febrile viral syndrome. The vector and syndrome tables are handy, and the disease descriptions contain sufficient information for preliminary consideration. However, this is not an exhaustive guide for clinical care and, as pointed out by the authors, does not obviate consultation of a more substantial reference or an expert in the management of a specific disease. Limited attention is given to isolation and infection-control recommendations. The CD ROM is a convenient feature, for example, for field use; however, more informative disks that accompany larger virology texts would probably have greater utility in such a setting.

